# Acceptability of prehabilitation for cancer surgery: a multi-perspective qualitative investigation of patient and ‘clinician’ experiences

**DOI:** 10.1186/s12885-023-10986-0

**Published:** 2023-08-11

**Authors:** Rachael Powell, Amy Davies, Kirsty Rowlinson-Groves, David P French, John Moore, Zoe Merchant

**Affiliations:** 1 Manchester Centre for Health Psychology, Division of Psychology and Mental Health, School of Health Sciences, University of Manchester, Manchester, UK; 2 Present Address: Division of Nursing, Midwifery and Social Work, School of Health Sciences, University of Manchester, Manchester, UK; 3 GM Active, Salford Community Leisure, Salford, UK; 4 Department of Anaesthesia, Manchester Royal Infirmary, Manchester University NHS Foundation Trust, Manchester, UK; 5 Greater Manchester Cancer Alliance, The Christie NHS Foundation Trust, Manchester, UK; 6 Division of Diabetes, Endocrinology and Gastroenterology, School of Medical Sciences, University of Manchester, Manchester, UK; 7 Present Address: North West Lung Centre, Lung Cancer and Thoracic Surgery Directorate, Wythenshawe Hospital, Manchester University NHS Foundation Trust, Manchester, UK

**Keywords:** Cancer, Surgery, Prehabilitation, Recovery, Acceptability, Physical fitness, Physical activity, Adherence, Engagement, Compliance

## Abstract

**Background:**

‘Prehabilitation’ interventions aim to enhance individuals’ physical fitness prior to cancer treatment, typically involve exercise training as a key component, and may continue to support physical activity, strength, and fitness during or after treatment. However, uptake of prehabilitation is variable. This study investigated how patients from diverse socio-economic status groups perceived an exemplar prehabilitation and recovery programme, aiming to understand factors impacting acceptability, engagement and referral.

**Methods:**

This research was conducted in the context of the Prehab4Cancer and Recovery Programme, a prehabilitation and recovery programme available across Greater Manchester, UK. Qualitative, semi-structured phone/video-call interviews were conducted with 18 adult patient participants referred to the programme (16 ‘engagers’, 2 ‘non-engagers’; half the sample lived in localities with low socio-economic status scores). An online questionnaire with free-response and categorical-response questions was completed by 24 ‘clinician’ participants involved in referral (nurses, doctors and other staff roles). An inductive, multi-perspective, thematic analysis was performed, structured using the Framework approach.

**Results:**

Discussing and referring patients to prehabilitation can be challenging due to large quantities of information for staff to cover, and for patients to absorb, around the time of diagnosis. The programme was highly valued by both participant groups; the belief that participation would improve recovery seemed a major motivator for engagement, and some ‘clinicians’ felt that prehabilitation should be treated as a routine part of treatment, or extended to support other patient groups. Engagers seemed to appreciate a supportive approach where they did not feel forced to do any activity and tailoring of the programme to meet individual needs and abilities was appreciated. Initial engagement could be daunting, but gaining experience with the programme seemed to increase confidence.

**Conclusions:**

The prehabilitation programme was highly valued by engagers. Introducing prehabilitation at a challenging time means that personalised approaches might be needed to support engagement, or participation could be encouraged at a later time. Strategies to support individuals lacking in confidence, such as buddying, may be valuable.

**Study registration:**

The study protocol was uploaded onto the Open Science Framework 24 September 2020 (https://osf.io/347qj/).

**Supplementary Information:**

The online version contains supplementary material available at 10.1186/s12885-023-10986-0.

## Background

Surgery is often part of treatment for individuals with cancer, but entails risks and post-operative complications frequently occur [1]. Higher levels of physical activity and functional capacity before surgery have been associated with better post-operative outcomes such as lower risk of post-operative complications [2, 3]. Other potential benefits of physical activity include reduced risk of future cancer and improved physical functioning and quality of life [4, 5].

There is increasing interest in ‘prehabilitation’ interventions designed to enhance physical functioning prior to treatment such as surgery. Physical fitness training to enhance cardiovascular and musculoskeletal health is often a key component of such programmes, alongside elements such as nutritional and mental health support [6]. Programmes may also include ‘rehabilitation’ elements, aimed to help patients to recover from cancer treatment and to reduce the risk of health conditions including future cancer diagnoses [6].

Systematic reviews of prehabilitation interventions involving exercise training suggest prehabilitation programmes show promise, albeit with some inconsistencies. Reviews of perioperative exercise training interventions have suggested such training to be associated with reduced risk of post-operative complications and reduced length of hospital stay in people with lung cancer [7, 8], albeit not for people with urologic and gastrointestinal cancers [9, 10]. A review of trials of prehabilitation programmes including exercise with abdominal cancer patients suggested no impact on post-operative complications but did see a reduction in length of hospital stay for intervention groups [11]. There appear to be other benefits of exercise training around surgery. For example, improvement in cardiorespiratory fitness was associated with exercise training prior to urologic cancer surgery, and exercise training interventions delivered post-operatively to people who have received surgery for lung cancer were associated with increased exercise capacity and leg muscle strength [9, 12].

Some inconsistencies in findings may result from variation in intervention content, intensity and frequency of delivery [7, 9, 11]. An important further issue is uptake: the extent to which individuals engage with, and continue participating in, programmes. One systematic review that examined evidence in 22 included studies regarding impact of prehabilitation on individuals receiving surgery for abdominal cancer, noted that rates of participants declining to take part in trials of prehabilitation ranged from 0 to 82% [11].

Whilst these figures represent engagement in trials of prehabilitation rather than in prehabilitation per se, perceptions of prehabilitation programmes may have influenced willingness to participate. Understanding the acceptability of interventions is recognised as a key aspect of intervention development and evaluation [13]. Acceptability can be conceived as “a multi-faceted construct that reflects the extent to which people delivering or receiving a healthcare intervention consider it to be appropriate, based on anticipated or experienced cognitive and emotional responses to the intervention” [14] (p4). Further, it is possible that an individual may regard an intervention as beneficial and acceptable, but there may yet be barriers affecting their participation, especially in the context of a cancer diagnosis.

Research into factors affecting engagement with prehabilitation has found that transportation challenges is a commonly reported barrier [9, 15–18]. Time pressures have also been noted, resulting from e.g. multiple medical appointments and individuals having pre-existing commitments [15, 16, 18, 19]. Qualitative research has suggested that tailoring programmes to individuals’ needs and preferences seems to be viewed favourably, such that individuals would be able to take part, without finding programmes overly challenging, whatever their ability level [15, 19, 20].

In research conducted to date, the socio-economic status of participants is rarely reported. Individuals living in lower socio-economic status (SES) areas are less likely to conduct recommended levels of physical activity in general than those in in higher SES areas, and individuals in ‘low-income households’ are less likely to carry out sports or exercise than those in ‘high-income households’ [21–23]. Problems impacting participation in prehabilitation may be particularly pertinent to individuals in lower SES areas [24].

Individuals are typically referred into prehabilitation by members of their care team. As such, clinical staff are ‘gatekeepers’ and whether or not they refer patients may be influenced by perceptions about what interventions are suitable for whom [25]. Investigating health professionals’ experiences of the referral process, and perceptions of prehabilitation, is valuable in understanding barriers impacting referral. Health professionals also have valuable experience which may provide insights regarding why individuals decline to take part in prehabilitation.

The present study aimed to understand how patients with colorectal, lung or oesophago-gastric cancer perceived a prehabilitation and recovery programme and to identify facilitators and barriers to engagement, whilst ensuring inclusion of individuals from lower SES areas. It also aimed to understand barriers and facilitators associated with referring patients to the programme, and the perspectives of healthcare staff on prehabilitation. A qualitative approach was used, seeking to gain an in-depth, meaningful understanding of patient and clinician experiences.

## Methods

### Design

Single, semi-structured, qualitative phone or video call interviews were conducted with patients recruited from a cohort who were referred to a prehabilitation programme in Greater Manchester prior to cancer surgery. ‘Clinician’ participants (healthcare professionals or other NHS (National Health Service) staff members involved in referral processes) completed an online questionnaire.

### Setting

Greater Manchester (GM) is a combined authority area in North West England with a population of approximately 2.68 million people, and has high levels of deprivation across its ten constituent metropolitan boroughs [26]. The GM Cancer Alliance Prehab4Cancer and Recovery (P4C) Programme aims to provide supported exercise to people with colorectal, lung or oesophago-gastric cancer before, during and after treatment, with nutritional status and mental wellbeing also assessed and supported [6]. These three patient groups were selected as the first to be offered the P4C Programme because of the evidence base and pre-existing prehabilitation interest and support from the specific healthcare teams working within the local cancer alliance [6]. Details of the programme are provided in Table 1. A quantitative evaluation reported positive outcomes including reduced post-operative length of stay, reduced readmissions and physical activity improvements [27]. Both pre- and post-surgery elements were completed by 73% of referred patients [27].

**Table 1:** Features of the Greater Manchester Cancer Alliance Prehab4Cancer and Recovery Programme (P4C Programme)

Feature	Description
**Programme deliverer**	GM Active: a collaboration of the public leisure providers in Greater Manchester.
**Staff involved in referral** (refering patients themselves / involved in referral decision-making)	Healthcare staff across Greater Manchester NHS Trusts including: doctors (e.g. Surgeons, Oncologists, Anaesthetists), nurses (e.g. Cancer Nurse Specialists or ERAS nurses), Allied Health Professionals (e.g. Physiotherapists, Dieticians) and support staff (e.g. Cancer Care Co-ordinators).
**Pre-surgery intervention**	• Patients’ fitness assessed, allocated to ‘universal’ or ‘targeted’ pathway.
	• ‘Targeted’ pathway: for individuals with lower fitness/greater support needs. Thrice-weekly sessions, supervised by Exercise Specialists
	• ‘Universal’ pathway: Relatively independent, self-managed sessions in leisure facilities close to patient’s home, with monitoring by Exercise Specialists and support if needed.
	• Both pathways: free gym membership.
**Post-surgery intervention**	• Commence rehabilitation phase 6 weeks post-surgery.
	• Personalised exercise programme, focus: post-treatment recovery.
	• 12 more weeks of free gym membership.
	• After March 2020 (COVID restrictions): remote provision. Initially: phone check-ins and assessments; exercise programmes and bands posted to patients. Later: included online group exercise via video call and MyZone heart rate monitors enabling Exercise Specialists to monitor exercise intensity remotely.

ERAS = Enhanced Recovery After Surgery

### Participants

#### Patient participants

Inclusion criteria were: aged over 18 years; able to speak and understand English; received surgery for cancer May 2019 - March 2020; referred to the P4C Programme. These individuals were offered face-to-face pre-surgical support, and face-to-face or remotely provided post-operative support. Individuals were excluded if they were deemed unsuitable for the P4C Programme in baseline assessment or if they received a change in diagnosis. Individuals were also excluded if there was insufficient time for them to take part in the P4C Programme before surgery; individuals were included if there was sufficient time at the point of referral for the patient to be invited for baseline assessment.

Participants were purposively sampled with the aims of including individuals who did, and did not, engage with the P4C Programme, and of including participants from low SES areas. A ‘non-engager’ was someone who was referred but did not attend the baseline assessment, or who attended the baseline assessment but did not then take part in the exercise programme. We aimed to conduct approximately 15 interviews with both ‘engagers’ and ‘non-engagers’ to gain a range of perspectives whilst being able to obtain a deep understanding of issues of importance to the participants.

#### ‘Clinician’ participants

Healthcare professionals or other NHS staff members involved in the referral process for the P4C Programme were eligible to take part. Approximately 200 staff were involved in referral; a 30% response rate would yield a sample of 60.

### Procedure & data collection

#### Patient participants

KRG identified individuals in the P4C Programme database who met study inclusion criteria. Study invitation packs were mailed to the home addresses of eligible patients, including all eligible ‘non-engagers’, by GM Active staff October 2021 - December 2021. Initially, individuals living in the three most deprived deciles according to the English Indices of Deprivation online tool were invited to take part [26]. Individuals who received surgery most recently were then invited. Individuals were asked to contact the researcher (AD) by telephone, text message or email if they were interested in participating. Individual interviews were conducted by phone or video call (Zoom) by AD, a university-based researcher independent of the P4C Programme.

Interviews were guided by an interview schedule developed by the research team and reviewed by public involvement contributors (Appendix A, Supplementary Material 1). Participants were asked about their experience of referral, their perceptions of the programme and barriers and facilitators to participating. For ‘engagers’, experiences of taking part were discussed. The Theoretical Framework of Acceptability (TFA) was used to structure later interview questions to ensure that theoretically relevant aspects of acceptability were covered within the interview [14]. The TFA proposes that ‘acceptability’ is multi-factorial, comprised of seven theoretical components including Affective Attitude, Burden, Perceived Effectiveness and Self-Efficacy [14]. Participants were also asked for demographic information, postcode (to establish Index of Multiple Deprivation score), and how much they engaged with the P4C Programme. Interviews were audio-recorded, and field notes made following interviews.

#### ‘Clinician’ participants

Recruitment and survey completion was open November 2021 - January 2022. Emails promoting the study were sent by ZM to key individuals including clinical leads at hospital sites and representatives of clinical referring teams. Those contacted were asked to cascade emails to all staff involved in P4C referral. The study was also advertised on an online forum and staff were reminded of the study using Twitter. Adverts contained a weblink to the study information and survey.

The online survey was developed by the research team, including clinician members, and hosted on SelectSurvey. A mixture of categorical response options and free-response boxes were used to minimise burden for busy NHS staff whilst also gaining insight into staff perspectives (Appendix B, Supplementary Material 2). Topics covered included: experiences and thoughts related to referring patients to the programme, perceptions of the programme, and what they thought might help patients to take part.

### Analysis

Thematic analysis was conducted, aiming at identifying and understanding ‘patterns’ in the data [28, 29]. We sought to gain a deep and meaningful understanding of issues discussed within the dataset, whilst ensuring that our interpretations were based on, and supported by, the data. An inductive, data-driven approach was taken to understand the experiences and perceptions of participants across both patient and clinician groups [see Appendix C (Supplementary Material 3) for analysis details]. A multi-perspective analysis was conducted; the patient and ‘clinician’ datasets were brought together during analysis so that issues could be considered from the viewpoints of both patients and healthcare staff. The analysis was structured using the Framework approach [29, 30]. The Framework approach provides a strategy for managing data throughout the analysis process. It involves the use of matrices, or ‘charts’, in which data are summarised, aiding the interrogation and understanding of the dataset [29, 30]. Responses to categorical survey questions were summarised numerically (Appendix D, Supplementary Material 4). The ‘clinician’ sample was not expected to be representative of the population, so this information was used descriptively, in an exploratory manner, as an aid to understanding clinicians’ experiences and their free-text responses within the qualitative analysis. RP and AD led the analysis, supported by all other authors.

## Results

To maintain anonymity, participants are identified by letters (patients) or numbers (clinicians) and contextual information by which individuals might be identified is removed from quotes. On occasion, the participant identifier is removed to minimise risk of identification through contextual information.

### Participant details

#### Patient sample

Invitation packs were mailed to 105 ‘engagers’ and 103 ‘non-engagers’. Twenty-five responses were received. Two individuals declined to take part; two did not meet study inclusion criteria; two intended recipients were deceased; one cancelled a planned interview for health reasons. Eighteen interviews were conducted October 2020 - January 2021.

Participant characteristics are detailed in Table 2. Engagers’ participation in the P4C Programme varied: pre-operatively, reported engagement ranged from 0 to 1 exercise sessions in total to regular attendance thrice weekly. Most reported regular post-operative engagement, ranging from attending one session a week to carrying out exercises ‘most days’; for some, participation was interrupted by ill health.

**Table 2:** Patient sample characteristics (n = 18)

Characteristic	Participants/participant information
Gender	
Female	9
Male	9
Age	Median 68.5 years (range 40s to 80s)
Ethnic group	
White British	16
Other ethnic group	2
Socio-economic status^†^	
IMD score 1–3	9
IMD score 4–6	5
IMD score 7–10	4
Diagnosis	
Bowel/colon cancer	9
Lung cancer	7
Oesophago-gastric cancer	2
Employment	
Retired	13
Employed	3
Unemployed	2
Participation in P4C programme	
Engager	16
Non-engager	2
Interview medium	
Phone	15
Videocall	3
Interview duration	Median 43 min (range 29–99 min)

^†^IMD = Index of Multiple Deprivation. IMD score 1 = most deprived locality; IMD score 10 = least deprived locality

#### ‘Clinician’ sample

Twenty-five individuals completed the online survey during November-December 2020. One did not meet inclusion criteria, leaving 24 eligible responses. Participant characteristics are described in Table 3.

**Table 3:** ‘Clinician’ sample characteristics (n = 24)

Characteristic (n reporting information)	Participants/participant information
NHS role (22) ^†^	
Nurse	11
Doctor	7
Other	4
Time since qualification (20)	Median 19.5 years (range < 5 to > 35 years)
Time involved in P4C referral pathway (22)	Median 18 months (range < 5 to > 35 months)
Role in referral pathway (22; some had > 1 role)	
Directly refer patients	16
Input into referral decision	10
Introduce patients to the programme	2
Age (19)	Median 44 years (range 30s to 50s)
Gender (20)	
Female	16
Male	4
Ethnic group (21)	
White/White British	20
Other ethnic group	1

^†^ NHS roles are grouped into these broad categories to avoid risk of identification of individuals. At least 9 different roles were represented within the sample

#### Impact of COVID-19 on recruitment

The COVID-19 pandemic impacted recruitment of patient and ‘clinician’ participants. The study recruitment period coincided with restrictions on working practice and capacity for NHS staff (potential study participants) and the research team. Nevertheless, the target sample size for ‘engagers’ was achieved. Recruitment of ‘clinician’ participants was particularly restricted: the researcher was unable to visit referring teams and it was not appropriate to send some planned electronic reminders because of extreme workload pressures. Whilst the ‘clinician’ sample size was smaller than anticipated, we were pleased that so many completed the online survey in the circumstances. The range of roles participants reported suggest that we are likely to have captured a broad cross-section of views, and thoughtful responses were received, with 22 of the 24 ‘clinician’ respondents writing free-text responses rather than only selecting categorical response options.

### Analytical findings

Five analytical themes related to acceptability were developed: A challenging time; Perceived value of the programme; Fitting with individuals’ needs; Impact of previous exercise experience; and Accessibility. As the findings related to Accessibility (particularly issues around transport and time commitments) are in line with previous findings [9, 15–19], we report the first four themes here. A further important area identified was the emotional well-being impact of participating in the programme; as this was not directly related to the focus on the present paper on acceptability, this topic is fully reported elsewhere [31].

#### A challenging time

Patients were typically referred to the prehabilitation programme around the time of diagnosis, when they were likely to have a lot to process:

*Patients often have a lot to contend with when first diagnosed. Additional appointments and commitments can cause confusion and stress. They can often receive 2, 3 or 4 hospital appointments in a week and feel fatigued by repeated contacts. (Clinician 14)*

One non-engager spoke about feeling overwhelmed with their diagnosis and the requirements associated with it, and seemed to feel that it was not a good time to have been approached about the programme:

*One day I’m going for [a hospital appointment], the next day I’m going to go and meet this guy to do some exercise and the next day I’m having [another hospital appointment], I was like, what, what. In the middle of all of that I’ve got to hold down a job […] But how it was delivered was like just go away and leave me alone, I’m not up for all of this at the moment [a short time later in interview:] And I felt exhausted, I felt mentally exhausted. (Patient G, non-engager).*

Engager participants seemed to find the approach strategies used acceptable: provision of initial brief information, followed up with more detailed discussion with programme staff:

*I think it [the leaflet] was about right. If you go into too much detail it can end up putting people off. (Patient O, engager)*

For some, therefore, brief information at the time of diagnosis seemed acceptable and appropriate: it provided an introduction without leading them to feel overwhelmed.

Many ‘clinician’ participants identified ‘forgetting to mention Prehab4Cancer’ as something which can make it difficult to refer patients to prehabilitation (Appendix D), and it seemed that having a lot to cover in an appointment may contribute to this. Nevertheless, it seemed that ‘clinicians’ viewed discussing prehabilitation with patients as important, and identified strategies to address forgetting:

*Sometimes patients are given a lot of information at the time of diagnosis and you have good intentions of mentioning prehab but forget. If this is the case we will try and contact patients after to discuss. (Clinician 13)*

Staff needed to approach individuals about prehabilitation close to the time of diagnosis in order to ensure that patients would have time to engage with the programme prior to treatment. Some discussion occurred in both patient and ‘clinician’ responses about the timing of the prehabilitation programme relative to diagnosis and treatment. A short time to surgery could affect whether or not they could be referred:

*If patients come late for decision to surgery and they have less than 2 weeks to be operated upon to avoid target breach, it is not feasible to refer them to the program. (Clinician 24)*

One non-engager seemed willing to learn more about the programme, but having surgery brought forward made it impossible for them to attend their planned assessment with programme staff:

*It was when I was waiting for the operation and I arranged to go to a meeting but the operation was brought forward and it overlapped, so I couldn’t go to the prehab. (Patient P, non-engager)*

Some patients indicated perceived value in having a longer time to do prehabilitation prior to surgery:

*it depends from when the prehab team gets hold of them to when they’re due for surgery. And I think the bigger that gap then there’s more chance of them getting fitter. (Patient J, engager)*

In response to being asked what they would like to see the P4C Programme do differently, one ‘clinician’ suggested delaying cancer treatment to ensure more time was available for prehabilitation:

*have longer to provide it i.e. step some patients off the cancer treatment pathway to give longer periods of prehab before surgery (Clinician 12).*

Nevertheless, despite some patients valuing having time for prehabilitation pre-treatment, it is not clear whether patients would support delaying treatment to achieve this. For example, Patient A seemed keen to move through surgery as quickly as possible:

*I just wanted to get it over and done with. The sooner I’d have it done, the quicker I’d be home and I’d be on the mend. (Patient A, engager)*

#### Perceived value of the programme

Engager participants tended to use language which indicated that the programme was very highly valued, with some expressing gratitude or a sense of good fortune in its being available to them:

*I’m so lucky to have been part of that (patient J, engager)*

Most ‘clinicians’, when asked how valuable they thought taking part in the programme is for patients, indicated ‘extremely valuable’ or ‘very valuable’ (Appendix D). When asked what they perceived the benefits of the programme to be for patients, most of the available options were endorsed by most ‘clinician’ participants (improved fitness, quicker recovery post-surgery, fewer complications post-surgery, improved long-term physical activity levels, improved long-term health or fitness and meeting people). There were also free-text comments demonstrating high perceived value of the programme, for example:

*all patients […] have nothing but praise for the prehab scheme and the staff that provide the service* [and later:] *I believe the prehab scheme has been extremely beneficial for our patients and the team do a fantastic job (Clinician 22).*

This sense of value was highlighted by some ‘clinicians’ wishing to see the service extended to include additional patient groups:

*We would love our benign patients to have the same service they would absolutely benefit massively. (Clinician 15)*

This high perceived value of the programme might be why, despite the challenges of introducing the prehabilitation programme, ‘clinician’ participants almost all reported that they ‘always’ or ‘usually’ referred eligible patients to the programme. ‘Clinicians’ also reported that patients ‘rarely’ decline (Appendix D). ‘The patient not wishing to be referred’ was the most common patient characteristic selected as leading to non-referral. It is therefore important to understand patients’ thoughts about prehabilitation, and how such perceptions might influence engagement.

Some patient participants seemed to have been highly positive about the programme from the start.

*I actually thought it was a great idea. And a real boon to get your fitness level up before an operation. Because it – well it increases your survival rate and it improves your recovery time afterwards. (Patient O, engager)*

Understanding that taking part in prehabilitation could improve recovery seemed a major motivating factor for patients, noted also by ‘clinician’ participants:

*I think most patients are nervous about the prospect of surgery but are happy to partake in a programme that will help them to recover quicker afterwards (Clinician 23)*

One of the non-engager participants did not seem to perceive benefits to participating in the programme:

*Interviewer: How effective did you feel that the exercise programme would have been in preparing you for surgery? Do you have any thoughts on that?*

*Patient G (non-engager): Not at all.*

*Interviewer: and why’s that?*

*Patient G: Because it was going to be a major operation. I knew that and I knew that I would have physio care afterwards*

This individual did not seem to find it plausible that taking part in prehabilitation would help them with recovery because it was major surgery and they anticipated receiving sufficient routine care to cope with it.

There were also engager participants who were initially sceptical of the programme:

*I thought it was a load of rubbish when I first started [laughs]. The idea of it I thought how’s that going to help, and then once I started doing it I realised how much better I felt and thought, yeah, this is my – that was it. (Patient E, engager)*

With hindsight, many engager participants felt themselves to be fitter and stronger as a result of having taken part in the programme. Some perceived that this helped ensure they were prepared for surgery, and attributed positive recovery trajectories to the programme:

*I was a bit naïve thinking that oh yes, after they’ve taken this tumour out, I’ll be fine, I’ll be able to whatever. And if I hadn’t had done the exercises, I don’t think my recovery rate would have been as good. (Patient R, engager)*

Some engagers appeared surprised by the extent to which exercises supported mobilisation post-surgery; it seemed that despite having engaged with the programme, until surgery happened, they had not fully appreciated how the programme could benefit their recovery.

Previous experience of surgery – whether as a patient themselves, or hearing about others’ experiences – seemed to influence perceptions around the value of taking part in the programme. A few engager participants reflected on their own or others’ previous experiences, for example:

*I thought it was good because I have a friend who’s had cancer […] He said the one piece of advice he’d give me was to be as fit as possible before going into the op. (Patient F, engager)*

On the other hand, previous own or vicarious experience seemed to influence both non-engagers’ perceptions that standard post-operative care would suffice:

*I didn’t think it really mattered that much because I’ve seen other people have the operation, […] I knew what was going to go on and the outcome afterwards. (Patient P, non-engager)*

The importance of understanding the programme’s benefits was emphasised by ‘clinicians’: several selected ‘language barrier’, ‘lack of understanding of the programme’ and/or ‘think programme will not benefit them’ as reasons for declining referral (Appendix D). ‘Education about the benefits of prehab’ as a strategy to help patients to take part was endorsed by many, with Clinician 16 commenting:

*We need to ‘sell’ the benefits of the program to the patients at the time of referral. In the same way they won’t take medications if they don’t realise the reason/importance of taking it.*

### Fitting with individuals’ needs

#### Patient choice/control

A small number of ‘clinician’ participants endorsed ‘Treatment options being restricted if they do not engage’ as a strategy to help people to take part, implying that options such as surgery could be limited to those who did participate. Some ‘clinicians’ reported that they describe the programme as part of routine treatment, or treat referrals to prehab in the same way that they would refer patients for other components of treatment. Treating prehabilitation as part of routine care seemed to be perceived to increase adherence: ‘*it needs to be described as part of their treatment’ (Clinician 15).*

However, treating prehabilitation as part of routine care, without carefully discussing it with patients and considering their views, could be counterproductive:

*And I just felt like it had all been organised and it’d all been given to me and no consideration has been given to, um, was that what I wanted? […] It was all kind of like pre-organised and it was like giving me a prescription, with you’re going to do that next, and that next, and that next, and that next, and all that. (Patient G, non-engager)*

[and later in the interview:]

*Interviewer: What do you think madeyounot take part?*

*Patient G: Um, it was the way it was presented to me, and it was given to me as this is something I’m going to have to do, rather than, would you like to do this.*

This individual seemed to be feeling overwhelmed and seeking to gain control over their situation, so if healthcare staff made assumptions about referral they may have contributed to the patient feeling put off prehabilitation.

Some engagers suggested that a lack of pressure within programme delivery was beneficial, that a supportive rather than directive approach was appreciated:

*It wasn’t kind of, you must do this, you must do that, it was all very much encouragement and support that kind of thing (Patient J, engager)*

*[the Exercise Specialist] said at any time you’re doing something and you want to stop, you just stop, […] you could just walk away just like that if you felt the need which I never did as it happened. (Patient E, engager)*

It seems that staff minimising pressure could paradoxically lead to patients feeling reassured and happier about taking part. Engagement also seemed supported by general positive experiences with programme staff at their initial assessment:

*He was really nice, really enthusiastic, I was certainly more than happy to go back and meet him again. (Patient H, engager)*

#### Tailoring to individual

The P4C Programme’s exercise prescriptions were designed to be tailored to individuals; this was noticed, and appreciated, by engager participants.

*it were all done professionally and you were assessed before you were given exercises, they were tailor made to your individual needs […] so yeah, no concerns at all. (Patient L, engager)*

This tailoring seemed to increase individuals’ confidence that they would be able to manage the programme. When asked why someone might not take part, one participant responded:

*no matter what’s wrong with you, your exercises are tailor made to suit your needs. […] what one person might be able to do another person might not, but you can still do a programme that’s bespoke. (Patient L, engager)*

Whilst individuals generally felt pushed to improve their fitness levels, they also perceived any pushing to be within the limits of what they could achieve, and so did not seem to feel daunted or put off by this:

*they built you up nice and slowly, you know, obviously once you got to a certain level they tried other things and built it up (Patient E, engager)*

A contrasting view of prehabilitation was offered by a participant who declined the programme:

*You know, it’s a one fit all, why does everybody have to have, um, exercises before they have their operation for a start off. What effect was it going to be for me? (Patient G, non-engager)*

This individual seemed to doubt the efficacy of the programme as a result of being unaware that the programme was individualised, with each individual being pushed to achieve an increase in fitness.

Some ‘clinician’ participants endorsed characteristics related to patients’ health or fitness as affecting referral; similarly, small numbers of ‘clinicians’ endorsed ‘poor health’, ‘frailty’, ‘mobility problems’ and ‘low fitness levels’ as reasons for patients declining referral (Appendix D). Free text comments suggested that ‘clinician’ participants could sometimes have concerns about patients’ physical suitability, and that they thought that patients’ perceptions of their health could impact whether or not they would take part:

*The main reason for not referring is due to concerns re medical fitness for community-based prehab (Clinician 16)*

*Some patients […] feel they won’t be able to manage (Clinician 1).*

Greater awareness of the tailoring of exercises to suit individuals’ levels/abilities could potentially encourage healthcare staff to recommend the programme, and encourage patients to participate.

### Impact of previous exercise experience

#### Developing typologies

Amongst ‘clinician’ responses, being ‘already physically fit or active’ was selected by some as a perceived reason for patients declining referral to the P4C Programme (Appendix D), and was also raised in free-text comments, e.g.:

*Patients may also be well informed and undertake regular exercise and [are] therefore less willing to proceed. (Clinician 14)*

Conversely, being unfamiliar with exercise was seen as a factor that could deter participation:

*Patients who are not used to exercise might shy away from the thought of attending a gym. Most with encouragement will attend for a look. (Clinician 3)*

Thus, from a ‘clinician’ perspective, exercise experience seemed a factor which could impact participation. Within patient participants, there seemed to be some pre-conceptions about whom the programme was for in terms of fitness:

*I think the idea, if I’m not mistaken, of this programme, is really geared more, I may be wrong, to couch potatoes, you know, who’ve got a major operation coming up, and to try and get them into some form of shape. (Patient I, engager)*

It seems possible, therefore, that someone’s exercise experience pre-diagnosis could affect likelihood of participation, either through affecting whether or not a patient thought the programme was for them, or through ‘clinicians’ having preconceptions about the appeal of the programme for them. We considered whether people with different previous experiences of exercising perceived the programme in similar or different ways. Three broad typologies were developed based on participants’ reported pre-surgical physical activity (Table 4).

**Table 4:** Typologies based on pre-surgical physical activity

Typology	Description	N
1. Little-exercisers	Could be active in day-to-day life but did little ‘exercise’ (‘exercise’ as “planned, structured and repetitive bodily movement done to improve or maintain one or more components of physical fitness”. [32] (p129). E.g. could be inactive, or could do some walking, have an active job, do gardening or housework, or do occasional exercise activity e.g. swimming.	7
2. Non-gym exercisers	Did exercise, but not in gym setting. E.g. could swim, cycle, attend exercise classes, go hillwalking, play tennis.	6
3. Gym-goers	Already regularly used gym facilities.	5

#### Confidence to take part

Some ‘clinician’ participants endorsed ‘lack of confidence in exercising’ as a reason for declining referral (Appendix D). Individuals could feel daunted about the prospect of taking part in the programme:

*a little bit nervous [laughs], I didn’t know what to expect and how I’d be pushed (Patient Q, engager, little-exerciser)*

*the first time that I went on me own I were a bit apprehensive, but then I were fine. (Patient L, engager, non-gym exerciser)*

*It was a bit daunting when she said weights and resistance bands and that lot, you know. And I thought, crikey, I’ve never done weights in my life, I’m not going to lift them and that, but they give you little ones, you know, different weights to start off with (Patient B, engager, little-exerciser)*

There seemed to be two aspects related to individuals’ confidence: confidence related to trying something in a new environment, with new people (particularly by oneself), and confidence around how they would cope with the exercises.

Having exercise experience did not necessarily lead to confidence in exercising; it seemed that experienced exercisers could value being supported to safely exercise in the context of cancer treatment:

*I suppose I was very conscious of the fact that if I didn’t do things properly I would end up with injuries […] it’s nice to train with somebody isn’t it who can keep an eye to make sure you’re doing things properly (Patient H, engager, gym-goer).*

The potential for a new experience, and specifically the gym environment, to be daunting was recognised by some ‘clinician’ participants:

*Also going to a gym can be intimidating which on top of dealing with a cancer diagnosis is a lot to deal with. I buddy patients up for peer support through their treatment and it really helps knowing they are not on their own. (Clinician 17)*

Many ‘clinicians’ endorsed ‘a buddy strategy’ as a way to help patients to take part in prehabilitation (Appendix D).

#### Perceptions of exercise programme

Across typologies, individuals seemed to derive enjoyment from taking part in the programme, and satisfaction in seeing improvement:

*I really enjoy it. I really enjoy doing the exercises. Like, it strengthens your muscles in your arms, your legs, your core, everything. (Patient B, engager, little-exerciser)*

*everything was a controlled challenge, and I do enjoy a challenge (Patient I, engager, gym-goer)*

Amongst engagers who carried out little exercise previously, there were some who, it seemed, had not expected to enjoy exercising in a gym:

*I must admit when I first went to the gym I thought, what the bloody hell am I doing here [laughs]? I must be mad!* [and later in interview:] *At first I disliked everything about it, but once I got into it I started enjoying it. (Patient A, engager, little-exerciser)*

It seems that, for some individuals, the experience of the programme was more positive than their expectations. A ‘clinician’ similarly reported that patients fed back that *‘I enjoyed it more than I thought I would’* (Clinician 3).

## Discussion

The present study found that a prehabilitation programme offered to individuals receiving cancer surgery was highly valued by engagers and ‘clinician’ participants, with the potential for enhancing surgical recovery a key motivator for participation. Individuals who did not engage seemed less convinced of participation’s benefits. The tailoring of exercise programmes to individual abilities and needs seemed to enhance acceptability to engagers, increasing confidence in ability to cope with exercises. Individuals with varied prior exercise experience, in a sample with varying SES backgrounds, seemed to find the programme acceptable, beneficial and enjoyable.

Introducing the programme seemed challenging for patients and staff around the time of cancer diagnosis: referral to prehabilitation needs to happen quickly if a patient is to participate in, and benefit from, an exercise programme prior to cancer surgery, but there was a lot for patients to manage and process at this time. Previous research has discussed how the pre-surgical time period can seem long – because of the urgency of having cancer surgery, but also short – because individuals have a lot to do, and a lot to process, before their surgery [33]. Increasing the time between cancer diagnosis and surgery could ease pressure on individuals, enabling them to both accomplish required or valued tasks and also engage in prehabilitation. However, it is not clear whether delaying cancer surgery for this purpose would be acceptable to patients, and a delay could impact treatment efficacy for some. Most engagers found receiving brief initial information from healthcare staff, followed by more in-depth discussion with the programme’s Exercise Specialists, acceptable and feasible, but others, already feeling overwhelmed, might need more time to come to terms with their situation before feeling ready to consider participation.

Some ‘clinician’ participants believed that the programme should be treated, and introduced, as a routine part of cancer treatment. However, it seemed that patients valued a supportive, rather than directive, approach to the exercise programme, and our companion paper discusses how some individuals may perceive benefit from having an aspect of treatment over which they feel they have control [31]. Healthcare staff may therefore need to ensure that, whilst communicating that participation is a routine part of cancer treatment and strongly encouraged, patients still feel they can decide whether to take part. Exercise Specialists had received training in Motivational Interviewing, a person-centred, supportive approach to helping people to change behaviours [34]. It might be useful for staff involved in referral to adopt such an approach where cancer patients are uncertain as to whether to accept the offer of prehabilitation.

There seemed to be some expectation, particularly amongst ‘clinician’ participants, that individuals who are either already engaged in exercise, or who carry out little exercise, might be unwilling to participate in an exercise programme. Elsewhere, it has been reported that some individuals thought that prehabilitation was not relevant to them due to pre-existing fitness levels [33]. However, we found that engagers with various exercise experience appeared to value participation. Regular exercisers seemed to appreciate expert advice and support through a serious health condition. Those who did little exercise could feel dubious about, and daunted by, the programme initially, but seemed to gain confidence and experience unexpected enjoyment. Healthcare staff can be ‘gatekeepers’ by not referring individuals for whom they believe prehabilitation might not be appropriate [25]. Assumptions about suitability might also less directly impact engagement, by affecting how staff present prehabilitation to patients.

The desire to support recovery from cancer treatment seemed of major importance for patients. This finding, from a largely older adult sample, contrasts with research focussed on physical activity provision for older adults in the general population. In the general older adult population, enjoyment of the activity, and valuing the activity of itself, seem to be important for engagement in physical activity, with health benefits of secondary concern [35]. In contrast, in the context of the immediate threat of cancer surgery, the health benefits of exercise seemed highly salient as a motivator for participation. Cross-sectional questionnaire and qualitative studies have similarly found optimising physical preparation and survival chances to be important motivators for participating in prehabilitation [18, 36]. However, to fully understand the contribution of motivational factors to engagement and adherence, a prospective research design is needed, where motivational variables (e.g. beliefs about benefits) are measured pre-intervention, and related to subsequent engagement. A prospective study investigating adherence to exercise in individuals receiving chemotherapy for breast cancer found that baseline motivational variables (including ‘instrumental attitude’ – e.g. how useful/beneficial the programme was perceived to be) did not predict adherence [37]. It is unclear whether the differential findings are due to differences in research design or treatment context.

### Strengths and limitations

This study’s purposive sampling strategy was effective in recruiting patient participants living in areas of varied SES, increasing the likelihood that our findings reflect experiences of people with varying levels of social deprivation. Despite challenges resulting from COVID-19 restrictions, we recruited sufficient participants to provide useful and varied insights across patient and staff groups. The varied roles of individuals included in the staff sample increased the potential range of perspectives of those who may act as gatekeepers to prehabilitation and/or have useful insights about patient and staff experiences. However, the sample lacked variation in some important respects. Only two ‘non-engagers’ participated, and the non-engagers we targeted for recruitment were those who had been referred to the P4C Programme; we did not approach individuals who declined referral. In order to understand non-engagement, it is important to gain broader perspectives of individuals who decline engagement at any stage. The remote recruitment approach used may have hindered participation of individuals who were less engaged in the programme, or a less intensive data-collection approach could be more manageable for some individuals.

Our sample was mostly White British, and research has identified that individuals from UK minority ethnic groups may experience additional barriers to exercise compared with White British individuals [38]. For example, mixed-sex exercise environments may not be acceptable, and language issues can also be a barrier [38]. In the present study, some ‘clinician’ participants identified ‘language barrier’ as a factor which could impact referral, emphasising the need to better understand issues impacting prehabilitation participation across ethnic groups. Further, whilst we successfully recruited individuals across localities with varying SES, it is also important that research examining experiences and outcomes in the context of cancer considers intersectionality between factors such as SES and ethnicity [39]. The need for diverse recruitment is an issue for this research field more widely [25]. Tools such as the Health Inequalities Assessment Toolkit (HIAT), designed to support researchers in designing, executing and disseminating research are likely to be valuable. The HIAT advocates strategies including involvement of individuals with ‘relevant lived experience’ throughout the research process [40].

### Implications

Healthcare staff should not make assumptions about willingness to participate based on individuals’ level of exercise experience. Individuals’ concerns and goals may differ, so individualised discussion and support around taking part may be valuable. Whilst initial brief information, followed up by more detailed discussions with prehabilitation staff, seemed acceptable to many individuals, some may wish to receive more initial information or the ability to consider prehabilitation at a time suitable for them.

The potential for prehabilitation to improve recovery from surgery seems a major motivator for engagement, and individuals who were initially sceptical seemed to value feeling physically fitter around surgery and recovery. It may be helpful to include testimonials from such individuals to help patients who are initially undecided about participation. Buddying may be a worthwhile strategy to facilitate initial engagement by individuals lacking in confidence.

Additional perspectives on prehabilitation are needed, particularly from a broader sample of non-engagers and individuals from minority ethnic groups. We would recommend involving individuals from such populations in designing future research recruitment strategies, and using tools such as the HIAT to ensure that inequalities are effectively considered [40]. It would also be useful for cancer prehabilitation trials to be systematically reviewed, with the aim of identifying factors associated with higher or lower rates of declining participation; this is another approach which might provide insights into issues impacting engagement in prehabilitation.

A further group which future research could usefully include is Exercise Specialists. These individuals had substantial involvement with patients who met them to discuss prehabilitation initially, and who continued on to participate in the programme. Exercise Specialists are likely to be able to share valuable experiences related to supporting engagement, and insights into patient experiences of considering and participating in prehabilitation.

## Conclusions

Participation in a prehabilitation and recovery programme was highly valued, with enhancing recovery from surgery seeming particularly important to patients and staff involved in referral. To ensure benefits reach a wide range of individuals, methods and timing of approach may need to be personalised. Research to fully understand perspectives of non-engagers and individuals from varied ethnic groups is needed.

### Electronic supplementary material

Below is the link to the electronic supplementary material.

Supplementary Material 1 - Appendix A

Supplementary Material 2 - Appendix B

Supplementary Material 3 - Appendix C

Supplementary Material 4 - Appendix D

## Data Availability

The datasets generated and analysed during the current study are not publicly available in order to maintain participant confidentiality, in line with ethical approval and participant consent. Restricted access only is permissible; contact the corresponding author for information.

## List of abbreviations

COVID-19: Coronavirus disease
ERAS: Enhanced Recovery After Surgery
GM: Greater Manchester
NHS: National Health Service
P4C Programme: The Greater Manchester Cancer Prehab4Cancer and Recovery Programme
REC: Research Ethics Committee
SES: Socio-Economic Status
TFA: Theoretical Framework of Acceptability
