# Supplementary material for: Acceptability of prehabilitation for cancer surgery: a multi-perspective qualitative investigation of patient and ‘clinician’ experiences
Source: BMC Cancer. 2023 Aug 11;23:744. doi: 10.1186/s12885-023-10986-0 (PMC10416438; doi:10.1186/s12885-023-10986-0)
Supplement: Supplementary file 1 — Supplementary Material 1 - Appendix A [file 12885_2023_10986_MOESM1_ESM.docx]

*Acceptability of prehabilitation for cancer surgery: A multi-perspective qualitative investigation of patient and ‘clinician’ experiences*

**Appendix A: Interview Schedule**

**First contact with the programme**

- Can you remember when you first heard about the Prehab Exercise Programme? Could you tell me about this?
  - How did you hear about it / Who mentioned it? (e.g. a nurse, GM Active staff) / When?
  - What did they tell you about it?
- What were your first thoughts about the programme?
  - What did you like about the idea?
  - What did you dislike about it?
    - Any worries or concerns?
- Did you take part in the Prehab Exercise Programme? (‘take part’ = at least attend an assessment)

**IF YES**

**Deciding whether to take part**

- What do you think prompted you to take part? (If question seems unclear: why do you think you took part?)
- What did you hope to gain from taking part? (How did you think it might benefit you?)
- Did you have any concerns about taking part? If yes, could you tell me about these?
- What information did you receive about the programme?
  - What did you think about this information?
  - (Prompt: Would you welcome any more information? About what? Or prefer less information?)

**Experiences of the Programme**

- Was the programme what you expected? (encourage to say more following response)
- What did you think about the programme?
  - What did you like about the Exercise Programme?
  - What did you dislike about it?
- Could you tell me about what you did within the programme?
  - E.g. first assessment? How many exercise sessions attended?
- How did you find the first assessment?
- How did you find the exercise sessions? – Could you tell me about the exercise sessions?
  - What did you enjoy/like about them?
  - What did you not enjoy/like?
- Were there any problems or difficulties in taking part in in the programme? (Prompts: Transport/accessing sessions? Health problems? Other commitments?)
- What would have made it easier for you to take part? (Prompts: Support to attend? Help with transport; Different location: Timing of sessions?)
- Did you take part in individual sessions or group sessions?
  - How did you find these?
  - What would your preference be?
- What did you think of your trainer?
- Did you miss any sessions? Do you recall why this happened?
  - If did not miss sessions – what made you keep going?
- Do you feel you benefited from the Exercise Programme at all?

IF BENEFITS: In what ways do you feel you benefited?

- Are there any ways in which you think the programme should be changed or improved?
- What do you think might help other people to take part?
- Why do you think some people might not take part?
- How did you feel about the amount of effort needed to take part in the Exercise Programme?
- How effective did you feel the Exercise Programme was in preparing you for surgery?
- How much did you feel you understood how the Exercise Programme might help you to prepare for surgery?
- How confident did you feel about taking part in the Exercise Programme?
  - About going along to sessions?
  - About doing exercises within sessions?
- How much did you feel the Exercise Programme stopped you from doing other things that you would have liked to have done before surgery?

**Nutrition and well-being support**

While exercise is a main part of the programme, some people also receive advice about nutrition or support for their well-being.

- Do you remember receiving advice about nutrition?
  - How did you find this advice? (e.g. how did you feel about it? How useful did you find it?)
- Did you receive support about coping or stress? Or to help you if your mood has been affected?
  - How did you find this support? (e.g. how did you feel about it? How useful did you find it?)

**Exercise/physical activity since surgery**

- Since having surgery, have you continued with exercise at all? Could you tell me about this? Has this been through the Exercise Programme?
- I’d like to ask you what you think about being physically active more generally.
  - Before surgery, what physical activity did you used to do outside of the Prehab Exercise Programme?
  - What do you like about physical activity generally? (What do you dislike?)
  - What kinds of physical activity do you enjoy?
  - Thinking about any kinds of physical activity that you enjoy, what is it that makes it enjoyable?
  - What kinds of physical activity do you not enjoy? – What is it that you don’t like?
  - What helps you to be physically active, or to take part in physical activity?
  - What can make it difficult for you to take part in physical activity?

**IF NO**

**Deciding whether to take part**

- What do you think made you not take part? (if seems unclear: Why was it that you did not take part?)
- Was there anything that made it difficult to take part? Could you tell me about this?
  - (Prompts: Transport/accessing sessions? Health problems? Other commitments?
- What would have made it easier for you to take part?
  - (Prompts: Support to attend? Help with transport? Different location?)
- What do you think you would have got out of taking part? (Prompt: How do you think it might have benefited you?)
- What information did you receive about the programme?
  - What did you think about this information?
  - (Prompt: Welcome any more information? About what? Or prefer less information?)
- How did you feel about the amount of effort that would have been needed to take part in the Exercise Programme?
- How effective did you feel the Exercise Programme would have been in preparing you for surgery?
- How much did you feel you understood how the Exercise Programme might help you to prepare for surgery?
- How confident did you feel you would be to take part in the Exercise Programme?
  - To go along to sessions?
  - To do exercises within sessions?
- How much did you feel the Exercise Programme might have stopped you from doing other things that you would have liked to have done before surgery?

**Exercise/physical activity outside of the programme (including post-surgery)**

- Before surgery, could you tell me about any exercise or physical activity that you used to do?
- How about now?
- I’d like to ask you what you think about being physically active more generally.
  - What do you like about physical activity generally? (What do you dislike?)
  - What kinds of physical activity do you enjoy?
  - Thinking about any kinds of physical activity that you enjoy, what is it that makes it enjoyable?
  - What kinds of physical activity do you not enjoy? – What is it that you don’t like?
  - What helps you to be physically active, or to take part in physical activity?
  - What can make it difficult for you to take part in physical activity?

**ALL**

**Preparation/support before surgery**

- Apart from the Prehab Exercise Programme, could you tell me about how you found the support for surgery offered to you?
  - E.g. meetings with doctors or nurses?
- Did you attend Surgery School?
  - How did you find this?
  - If not, do you remember being offered it? Do you remember why it was you didn’t (or weren’t able) to attend?
- What do you think was most important in helping you to prepare for surgery?
- What (if any) changes would you like to see?
  - Is there any support that you would have liked but did not receive?

**Finally: some quick questions about you!**

- What is your gender?
- What is your age?
- What is your occupation/job?
- What is your ethnic group?
  - Ask question in open way – if participant is uncertain, use below prompts [categories from UK 2011 census]:
    - White (White British, Irish, Gypsy/Irish Traveller, Other),
    - Mixed (White & Black Caribbean, White & Black African, White & Asian, Other multiple background),
    - Asian or Asian British (Indian, Pakistani, Bangladeshi, Chinese, Other)
    - Black or Black British (African, Caribbean, Other)
    - Other ethnic group (Arab, Other).
- What is your postcode? (full postcode required to estimate SES of locality – but this will be deleted from transcript)
- For your cancer surgery, what type of surgery did you have? (What part of the body was operated on?)
- [Check that the following points are clear (likely to have been covered above)]
  - Did you take part in the Prehab Exercise programme?
  - Did you attend an assessment with GMActive?
  - Do you remember how many exercise sessions you attended?

**Last question:**

- Is there anything else that you feel is important that we’ve not talked about?
