# Supplementary material for: Acceptability of prehabilitation for cancer surgery: a multi-perspective qualitative investigation of patient and ‘clinician’ experiences
Source: BMC Cancer. 2023 Aug 11;23:744. doi: 10.1186/s12885-023-10986-0 (PMC10416438; doi:10.1186/s12885-023-10986-0)
Supplement: Supplementary file 2 — Supplementary Material 2 - Appendix B [file 12885_2023_10986_MOESM2_ESM.docx]

*Acceptability of prehabilitation for cancer surgery: A multi-perspective qualitative investigation of patient and ‘clinician’ experiences*

**Appendix B: Clinician Online Survey Questions**

This questionnaire contains a mixture of tick-boxes and free response boxes. We would be very pleased to receive any details you are able to provide in explaining or elaborating on your responses.

Please avoid entering information which may lead to the identification of yourself or other people (e.g. people’s names etc).

**Part 1: Referring patients to the Prehab4Cancer Programme**

| **Question** | **Response options** |
| --- | --- |
| If you refer patients to Prehab4Cancer yourself:  When you see patients who are eligible for the Prehab4Cancer Programme, how often do you refer them to the programme? | Always / usually / sometimes / rarely / never / not applicable |
| If you input into referral decisions (e.g. at MDT meetings):  When patients who are eligible for the Prehab4Cancer Programme are discussed, how often does your team refer them to the programme? | Always / usually / sometimes / rarely / never / not applicable |
| What can make it difficult for you or your colleagues to refer eligible patients to Prehab4Cancer? (please tick all that apply)  Please elaborate on your answer if you are able to do so | - Not knowing who to refer the patient to - Would like to see further research demonstrating beneficial effects of prehabilitation - Lack of time within clinical appointment - Prehab4Cancer is not a priority - other important issues to discuss - Lack of confidence in talking to patients about physical activity - Forgetting to mention Prehab4Cancer - Feel that pre-surgery is not a good time to receive the Prehab4Cancer intervention - Other (please state)   Free response box |
| What would make it easier for you/your colleagues to refer eligible patients to Prehab4Cancer? | Free response box |
| What patient characteristics might make it less likely that an eligible patient is referred to Prehab4Cancer? (please tick all that apply)  Please elaborate on your answer if you are able to do so | - The patient does not wish to be referred - The patient is of older age - The patient is of younger age - Language barrier - Poor health - Comorbidities/complex medical issues - Frailty - The patient would not like the programme - The patient is obese or overweight - The patient has a low BMI or is underweight - The patient is already physically fit or active - The patient would not be able to get to the gym (e.g. limited access to transport or financial limitations) - The patient has mobility problems (e.g. they use a wheelchair or a mobility aid). - The patient is in paid employment - The patient is unemployed or retired - The patient has caring responsibilities - Other (please state)   Free response box |
| How often do patients decline to be referred? | Always / usually / sometimes / rarely / never |
| Why do you think patients decline to be referred? (please tick all that apply)  Please elaborate on your answer if you are able to do so | - Language barrier - Poor health - Frailty - Dislike the programme - Dislike physical activity - Already physically fit or active - Low fitness levels - Mobility problems - Unable to travel to the gym - Lack of confidence in exercising - Too busy - Other commitments - Not a priority - Lack of understanding of programme - Think programme will not benefit them - Other (please state)   Free response box |
| Are there any other reasons why eligible patients might not be referred to the Prehab4Cancer programme? | Free response box |
| How confident do you feel that you understand the Prehab4Cancer eligibility criteria?  Please elaborate on your answer if you are able to do so | Extremely confident / very confident / quite confident / not very confident / not at all confident  Free response box |

**Part 2: Your thoughts about the Prehab4Cancer Programme**

| **Question** | **Response options** |
| --- | --- |
| How valuable do you think taking part in Prehab4Cancer is for patients?  Please elaborate on your answer if you are able to do so | Extremely valuable / very valuable / quite valuable / not very valuable / not at all valuable  Free response box |
| What do you think the benefits of Prehab4Cancer are for patients? (please tick all that apply) | - Improved fitness - Quicker recovery post-surgery - Fewer complications post-surgery - Improved long-term physical activity levels - Improved long-term health or fitness - Meeting people - Other (please state) |
| What do you think the most important benefit of Prehab4Cancer is for patients? (please select a single option)  Please elaborate on your answer if you are able to do so | - Improved fitness - Quicker recovery post-surgery - Fewer complications post-surgery - Improved long-term physical activity levels - Improved long-term health or fitness - Meeting people - Other (please state)   Free response box |
| Have you received any feedback about Prehab4Cancer from patients?  If yes, what have patients said? | Yes / No  Free response box |
| What do you think the main reason(s) is/are for patients not taking part in Prehab4Cancer after they have been referred? | Free response box |
| What do you think might help patients to take part in Prehab4Cancer? (please tick all that apply)  Please elaborate on your answer if you are able to do so | - A buddy system - Travel assistance - Support with commitments (e.g. caregiving, work) - Correspondence with their employer - Education about benefits of prehab - Financial incentives - Treatment options being restricted if they do not engage - Other (please state)   Free response box |
| What would you like to see Prehab4Cancer do differently? | Free response box |

**Part 3: About you**

| **Question** | **Response options** |
| --- | --- |
| What is your profession? | Cancer Nurse Specialist / ERAS Nurse / General Nurse / Dietitian / Physiotherapist / Occupational Therapist / Speech and Language Therapist / Surgeon / Oncologist / Anaesthetist / Doctor (other) , Care Coordinator / Pathway Navigator / Other (please state) |
| Number of years since qualification (if applicable) | Box for numerical response |
| What is your role in the Prehab4Cancer Programme referral pathway? | Directly refer patients / Input into referral decisions e.g. through MDT meetings / Other (please state) (mark all that apply) |
| For how many months have you been involved in the Prehab4Cancer referral pathway? | Box for numerical response |
| What is your gender? | Female / Male / Other / Prefer not to say |
| What is your age? | Box for numerical response |
| What is your ethnic group? | A. White / White British  B. Mixed / Multiple ethnic groups  C. Asian / Asian British  D. Black / African / Caribbean / Black British  E. Other ethnic group |

**Final question:**

| **Question** | **Response options** |
| --- | --- |
| Is there anything else that you would like to add? | Free response box |

**Many thanks for taking part in this survey**
