# Supplementary material for: Acceptability of prehabilitation for cancer surgery: a multi-perspective qualitative investigation of patient and ‘clinician’ experiences
Source: BMC Cancer. 2023 Aug 11;23:744. doi: 10.1186/s12885-023-10986-0 (PMC10416438; doi:10.1186/s12885-023-10986-0)
Supplement: Supplementary file 3 — Supplementary Material 3 - Appendix C [file 12885_2023_10986_MOESM3_ESM.docx]

*Acceptability of prehabilitation for cancer surgery: A multi-perspective qualitative investigation of patient and ‘clinician’ experiences*

**Appendix C: Analysis & Coding Framework**

**Appendix Ci: Analysis**

Interviews were transcribed by a professional transcription service, and identifying details removed. Survey data were downloaded and organised into Word documents. Analysis was conducted from a critical realism ontological perspective, viewing reality as existing externally to individual experience, whilst regarding understanding of that reality as dependent on how individuals regard and interpret it.(1)

Thematic analysis was conducted, aiming at identifying and understanding ‘patterns’ in the data.(2, 3) We sought to gain a deep and meaningful understanding of issues discussed within the dataset, whilst ensuring that our interpretations were based on, and supported by, the data. An inductive, data-driven approach was taken, aiming to understand the experiences and perceptions of participants across both patient and clinician groups. A multi-perspective analysis was conducted; the patient and ‘clinician’ datasets were brought together during analysis so that issues could be considered from the viewpoints of both patients and healthcare staff. The analysis was structured using the Framework approach.(3, 4) Following the Framework approach provides the researcher with a strategy for managing data throughout the analysis process. It involves the use of matrices, or ‘charts’, in which data are summarised, aiding the interrogation and understanding of the dataset. As such, it is a tool which supports the analysis, rather than a theoretical framework influencing how the researcher thinks about the data.(3,4) The analysis process was led by RP and AD, with involvement of all team members.

The Theoretical Framework of Acceptability (TFA), developed by Sekhon et al. was used in developing the patient interview schedule.(5) Sekhon et al. recognised the importance of considering acceptability when developing and delivering health-related interventions and developed a definition of acceptability: ‘A multi-faceted construct that reflects the extent to which people delivering or receiving a healthcare intervention consider it to be appropriate, based on anticipated or experienced cognitive responses to the intervention’(p4).(5) The TFA proposes that acceptability is composed of seven factors: Affective Attitude, Burden, Ethicality, Intervention Coherence, Opportunity Costs, Perceived Effectiveness and Self-Efficacy.(5)

In the present study, the TFA did not drive the analysis process. Analysis was structured using the Framework approach.(3, 4) This is a flexible approach which does not constrain how the researcher thinks about the data or develops the analysis, and is a systematic and transparent process, enabling other research team members to follow steps and decisions taken by the analyst. Initial stages of analysis were conducted separately for the two participant groups with their different viewpoints, but later stages drew together the two datasets into the same analysis, allowing issues to be considered from different perspectives.

Analysis was conducted within Word documents and Excel spreadsheets. AD and RP familiarised themselves with the patient interview transcripts by reading and re-reading the interviews and noting potential issues of interest or importance. Both AD and RP coded the first three interviews: labels capturing ideas in interview content were developed. These codes were discussed and used to develop an initial draft coding framework. RP coded the remaining transcripts, adding to and developing the working framework, meeting weekly with AD to discuss developments. The resulting working framework, consisting of a hierarchical list of categories and sub-categories of key issues within the data, was then discussed with the full research team to gain wider perspectives on the analysis. Familiarisation and coding of clinician responses was conducted by RP, and an initial coding framework developed. The first nine surveys were coded and used to develop an initial coding framework; later surveys were re-read, with additional codes being identified and added to the coding framework as appropriate. The two coding frameworks from the patient and clinician data were then combined, producing a single coding framework reflecting ideas identified in the full dataset. AD read a sample of clinician surveys, and RP and AD discussed and refined the coding framework (Appendix Cii). RP applied this coding framework to all transcripts and surveys, ‘indexing’ the data.

Charts were developed, with framework categories in columns and participants in rows; cells contained the essence of participant responses, with line numbers, to ensure that the researchers could constantly track back to transcripts and not lose the context of ideas expressed. Charts were interrogated, seeking to understand the various perceptions voiced by participants within categories, and how categories related to each other. Some chart columns were re-ordered, bringing together related content and facilitating the final analysis stage of writing. RP worked back and forth between charts and interview transcripts to develop themes, review quotes in the context of the interviews and surveys, and increase understanding of issues. Throughout this process, there was intentional movement from management of the dataset towards an in-depth, interpretive analysis of the data. Preliminary findings were reviewed and discussed by the full research team, then further refined to produce the final analysis.

The online surveys included categorical, tick-box questions alongside free-response boxes. Responses to categorical questions were summarised numerically (Appendix D). The sample was not expected to be representative of the clinician population, so this information was used descriptively, in an exploratory manner, as an aid to understanding the study participants’ experiences and their free-text responses within the qualitative analysis.

Neither transcripts nor analysis documents were shared with participants for member checking to minimise participant burden, particularly given debate around the value of this practice.(6)

Patient quotes are presented as spoken. ‘Clinician’ quotes are presented as written, with spelling errors corrected. ‘[…]’ indicates where text has been removed in presenting quotes, to aid concise reporting.

**References**

1. Ormston R, Spencer L, Barnard M, Snape D. The foundations of qualitative research. In: Ritchie J, Lewis J, McNaughton Nicholls C, Ormston R, editors. Qualitative Research Practice: A Guide for Social Science Students & Researchers. London: SAGE Publications Ltd; 2014. p. 1-25.

2. Braun V, Clarke V. Using thematic analysis in psychology. Qualitative Research in Psychology. 2006;3:77-101.

3. Spencer L, Ritchie J, Ormston R, O'Connor W, Barnard M. Analysis: principles and processes. In: Ritchie J, Lewis J, McNaughton Nicholls C, Ormston R, editors. Qualitative Research Practice: A Guide for Social Science Students & Researchers. London: SAGE Publications Ltd; 2014. p. 267-93.

4. Ritchie J, Spencer L. Qualitative data analysis for applied policy research. In: Bryman A, Burgess RG, editors. Analysing Qualitative Data. London: Routledge; 1994. p. 173-94.

5. Sekhon M, Cartwright M, Francis J. Acceptability of healthcare interventions: An overview of reviews and development of a theoretical framework. BMC Health services Research. 2017;17:88.

6. Thomas DR. Feedback from research participants: Are member checks useful in qualitative research? Qualitative Research in Psychology. 2017;14:23-41.

**Appendix Cii: Coding Framework**

1. **Initial experiences**
   1. **Initial contact**
      1. How and when first contacted, impact of this
      2. Discussing at later/another appointment
   2. **Information provided**
      1. Thoughts on what received
      2. Language used
      3. Ability to gain further information
      4. Information recommendations to enhance participation
   3. **Experience of first meeting with Prehab team/ initial assessment)**
      1. Experience of initial assessment and early sessions
      2. Impact of first meeting/assessment on understanding
      3. Impact on apprehension and confidence regarding Prehab
      4. Relationship development with instructor
   4. **Process of engagement**
      1. Ease of process (patient perspective)
      2. Control over decision to take part
      3. Confidence issues/nerves; how overcome
   5. **Approaching patients (clinician perspective)**
      1. Ease/confidence in approaching
      2. How often refer
      3. Impact of role/involvement with prehab
   6. **Forgetting to refer**
      1. Quantity of material to cover
      2. Routine practice / lack of routine
      3. Forgetting despite investment/value/role in prehab
   7. **Strategies to address forgetting/missed referrals more generally**
   8. **Confidence in discussing issues with patients**
      1. Desire to know more about declining so can enhance approach
      2. Desire for more feedback
   9. **IT/form/referral process issues**
   10. **Declining referral (C perspective)**
       1. Experience of patients declining
       2. Reasons for non-referral by clinicians:
          1. Patient not wishing to be referred
          2. Other factors which could impact whether or not referred
2. **Prehab and Surgery/Recovery**
   1. **Understanding/beliefs in benefits related to surgery and outcomes**
      1. Initial understanding/expectations
      2. Impact of understanding/perception of benefits on motivation to take part, anticipated regret
      3. Persuading of importance/benefits/’selling’/encouragement as strategies to enhance participation.
      4. Staff perception of ‘physical’ benefits – including short-term and longer-term
   2. Framing of surgical/recovery experience in light of (non)engagement with Prehab
   3. **Perceived value of exercises seen to be directly related to recovery**
   4. **Increased confidence** - going in to surgery (fit enough to cope physically) and in mobilisation post-op
   5. **Obligation/responsibility**
      1. Obligation to take what’s offered, do one’s bit
      2. Belief must increase fitness to be allowed surgery; framing as part of treatment (vs optional); incentive if treatment options restricted if don’t
      3. Save NHS funds
   6. **Impact of own/others’ previous experience of surgery / rehabilitation on perceptions.**
3. **Features of Prehab perceived as important**
   1. **Instructor’s approach**
      1. Characteristics
      2. Instructor’s approach leading to enjoyment.
      3. Accessibility and approachability (and communication)
   2. **Tailoring**
      1. To individual – e.g. capabilities, preferences, needs
         1. Addressing concerns about abilities – strategy to enhance engagement
      2. To health condition/procedure
   3. **Being looked after**
      1. Regular monitoring
      2. Supported, safe
      3. Continuity, pre and post-op
   4. **Social aspects**
      1. Camaraderie, meeting people
      2. Concerns re others – e.g. feeling judged
      3. Groups vs individual preferences (and experiences)
         1. Prehab abilitation
         2. General physical activity
   5. **Fitness and strength**
      1. Keeping/regaining (general) fitness
      2. Perceptions about types of exercises
      3. Seeing progress (including value of assessment and monitoring)
      4. Helping coping with chemotherapy
   6. **General opportunities/benefits**
      1. Trying/learning new things
      2. Enjoyment of activities vs aspects disliked/challenging
      3. Creates structure of going to gym/exercising
   7. **Experience with MyZone app/heart rate monitor**
      1. Including social aspects
   8. **Dislike of programme / belief programme/PA not for them – reasons for declining/not referring.**
   9. **Concerns (including no concerns)**
4. **Social influence and support (Prehab and physical activity generally)**
   1. **Support from family (including practical support e.g. transport and emotional support)**
   2. **Valuing family being able to attend Prehab**
   3. **Exercising with others in family (or not)**
   4. **Encouragement to/from others (vs e.g. feeling ‘judged’)**
   5. **Gym staff support (including discouragement from non-Prehab staff)**
   6. **Buddying patients as strategy to enhance engagement**
5. **Prehab Psychological Support/Psychological benefits**
   1. **Explicit support within Prehab**
   2. **Implicit / inherent support within Prehab**
      1. Support from instructor
         1. Someone that can talk to about cancer (vs other people)
         2. Professional friend throughout journey (including value of contact postop) – sense looked after, cared for – pre and post-op
         3. Value of talking of what will be doing – positive outlook post-op
      2. Support from peers
      3. Positive focus – e.g. positive outlook, structure to day, something to look forward to
      4. Enhanced sense of control
      5. Increased confidence that when go to surgery, will be able to cope with surgery
      6. Impact of prehab on confidence generally
      7. Mental well-being benefits from exercise/taking part
      8. Post-op
         1. Mental health benefits when activities restricted
         2. Discharge from Prehab – worries/wanting to continue.
6. **Experience/perceptions of gyms and exercise**
   1. **Previous experience of exercise/gyms/physical activity/fitness**
   2. **Impact of previous experience on perceptions of Prehabilitation**
   3. **Perceptions of gym**
      1. Friendly, welcoming
      2. Prehab – opportunity to try gym; including gym membership – incentive
      3. Warm, dry, safe
      4. Off-putting aspects (including perceptions of others and others’ perceptions of you)
      5. Providing structure
      6. Thoughts re equipment
   4. **General motivations to exercise/be active vs barriers (e.g. weather)**
      1. **Prehab changing perceptions/behaviours (‘teachable moment’)**
   5. **Post-prehab (& post-op, if ambiguous) exercise**
      1. What continued with, what changed?
      2. Willingness to pay to continue physical activity
      3. Ongoing impact of health condition.
7. **Accessibility/resources**
   1. **Transport/location**
      1. How access – driving/driven, walking, bus
      2. Potential problems for people without car/transport issues/assistance
      3. Preference for local gym
   2. **Gym resources**
      1. Quality & capacity
      2. Free access/affordability
   3. **Managing around other commitments**
      1. Presence/lack of other commitments; support needed
      2. Flexibility – ability to juggle either Prehabilitation/other commitments
      3. Prioritisation/lack of time
      4. Online resources – impact on accessibility
   4. **Managing around health issues**
      1. Appointments
      2. Adaptation around limitations
   5. Financial issues; financial incentives
8. **Impact of Covid/Lockdown**
   1. **Gym vs home exercise**
      1. Distraction vs structure/focus of gym
      2. Accessibility and resources
      3. Concerns re infections
   2. **Perceptions of home exercise**
      1. Range of classes, choices, Teams vs phone contact and exercises
      2. Enjoyment, preferences, willingness to pay
      3. Perceptions of safety, injury risk
      4. Experience of group
      5. Value beyond cancer context – coping with lockdown
      6. Partners joining in
9. **Value of Prehab**
   1. Longer time pre-operation (and post-operation?)
   2. More widely available
   3. Number/range of benefits seen as important
   4. Expressing gratitude/praise
   5. Clinicians’ source of information regarding value
      1. feedback from patients
10. **Nutrition advice**
    1. **Information from Prehab and elsewhere**
    2. **Specialist dietary needs**
    3. **Clinicians – suggesting add nutritional/other support**
11. **Experience of preparation for surgery (generally)**
    1. **Information**
       1. Information needs/wants
       2. Factors affecting worries/concerns
    2. **Perceptions of care team support**
       1. Feeling listened to
       2. Trust in team
       3. Support from Macmillan
       4. Accessibility of HCPs, Macmillan
    3. **Support from family and peers**
    4. **Perceptions of surgery school**
       1. Ensuring know what to expect
       2. Increase confidence in ability to cope
       3. Meeting staff
       4. Breathing exercises
       5. Group issues
       6. Accessibility issues
       7. Value being able to bring family
12. **Informative content**
    1. **How much attended (pre and post op)**
    2. **Timing of operation and impact on what’s available**
13. **Miscellaneous**
    1. **Perception of self and ones attitude to life**
    2. **Perceptions of others**
       1. Perception of who Prehab is ‘for’
    3. **Experiences of cancer/treatment not covered elsewhere**
       1. Perception of chemotherapy
       2. Humour, importance of ‘little things’
       3. Access to hospital – transport issues
    4. **Research experiences**
       1. Suggestion that interviewer = part of care team
       2. Unhappy asked for demographic info (gender, age, ethnicity)
