# Supplementary material for: Acceptability of prehabilitation for cancer surgery: a multi-perspective qualitative investigation of patient and ‘clinician’ experiences
Source: BMC Cancer. 2023 Aug 11;23:744. doi: 10.1186/s12885-023-10986-0 (PMC10416438; doi:10.1186/s12885-023-10986-0)
Supplement: Supplementary file 4 — Supplementary Material 4 - Appendix D [file 12885_2023_10986_MOESM4_ESM.docx]

*Acceptability of prehabilitation for cancer surgery: A multi-perspective qualitative investigation of patient and ‘clinician’ experiences*

**Appendix D: Clinician Online Survey - Summary of quantitative responses (n=24)**

**Part 1: Referring patients to the Prehab4Cancer Programme**

| **Question** | **Response** | **N responses** |
| --- | --- | --- |
| If you refer patients to Prehab4Cancer yourself:  When you see patients who are eligible for the Prehab4Cancer Programme, how often do you refer them to the programme? | - Always | 14 |
|  | - Usually | 7 |
|  | - Sometimes | 0 |
|  | - Rarely | 0 |
|  | - Never | 0 |
|  | - not applicable | 3 |
| If you input into referral decisions (e.g. at MDT meetings):  When patients who are eligible for the Prehab4Cancer Programme are discussed, how often does your team refer them to the programme? | - Always | 10 |
|  | - Usually | 9 |
|  | - Sometimes | 1 |
|  | - Rarely | 0 |
|  | - Never | 1 |
|  | - not applicable | 2 |
| What can make it difficult for you or your colleagues to refer eligible patients to Prehab4Cancer? (tick all) | - Not knowing who to refer the patient to | 2 |
|  | - Would like to see further research demonstrating beneficial effects of prehabilitation | 0 |
|  | - Lack of time within clinical appointment | 3 |
|  | - Prehab4Cancer is not a priority - other important issues to discuss | 2 |
|  | - Lack of confidence in talking to patients about physical activity | 1 |
|  | - Forgetting to mention Prehab4Cancer | 12 |
|  | - Feel that pre-surgery is not a good time to receive the Prehab4Cancer intervention | 2 |
|  | - Other (please state)   ‘Other’ responses: not finding referral problematic (n=3); issues with electronic referral process (n=2); issues with when see patient in diagnostic process (n=2); short time to surgery (n=1); patient not wishing to take part (n=1); lacking information for referral (n=1). | 10 |
| What patient characteristics might make it less likely that an eligible patient is referred to Prehab4Cancer? (tick all) | - The patient does not wish to be referred | 21 |
|  | - The patient is of older age | 2 |
|  | - The patient is of younger age | 3 |
|  | - Language barrier | 9 |
|  | - Poor health | 6 |
|  | - Comorbidities/complex medical issues | 9 |
|  | - Frailty | 7 |
|  | - The patient would not like the programme | 2 |
|  | - The patient is obese or overweight | 0 |
|  | - The patient has a low BMI or is underweight | 0 |
|  | - The patient is already physically fit or active | 4 |
|  | - The patient would not be able to get to the gym (e.g. limited access to transport or financial limitations) | 5 |
|  | - The patient has mobility problems (e.g. they use a wheelchair or a mobility aid). | 7 |
|  | - The patient is in paid employment | 2 |
|  | - The patient is unemployed or retired | 1 |
|  | - The patient has caring responsibilities | 6 |
|  | - Other (please state)   ‘Other’ response: *‘Hearing difficulties […]’* | 1 |
| How often do patients decline to be referred? | - Always | 0 |
|  | - Usually | 0 |
|  | - Sometimes | 5 |
|  | - Rarely | 15 |
|  | - Never | 1 |
| Why do you think patients decline to be referred? (tick all) | - Language barrier | 5 |
|  | - Poor health | 4 |
|  | - Frailty | 3 |
|  | - Dislike the programme | 3 |
|  | - Dislike physical activity | 6 |
|  | - Already physically fit or active | 6 |
|  | - Low fitness levels | 2 |
|  | - Mobility problems | 2 |
|  | - Unable to travel to the gym | 7 |
|  | - Lack of confidence in exercising | 7 |
|  | - Too busy | 4 |
|  | - Other commitments | 7 |
|  | - Not a priority | 7 |
|  | - Lack of understanding of programme | 3 |
|  | - Think programme will not benefit them | 3 |
|  | - Other (please state)   ‘Other’ response: *‘Can’t find the ‘headspace’ amongst processing diagnosis’* | 1 |
| How confident do you feel that you understand the Prehab4Cancer eligibility criteria? | - Extremely confident | 9 |
|  | - very confident | 9 |
|  | - quite confident | 4 |
|  | - not very confident | 2 |
|  | - not at all confident | 0 |

**Part 2: Your thoughts about the Prehab4Cancer Programme**

| **Question** | **Response options** | **N responses** |
| --- | --- | --- |
| How valuable do you think taking part in Prehab4Cancer is for patients? | - Extremely valuable | 16 |
|  | - very valuable | 4 |
|  | - quite valuable | 3 |
|  | - not very valuable | 0 |
|  | - not at all valuable | 0 |
| What do you think the benefits of Prehab4Cancer are for patients? (tick all) | - Improved fitness | 22 |
|  | - Quicker recovery post-surgery | 23 |
|  | - Fewer complications post-surgery | 17 |
|  | - Improved long-term physical activity levels | 18 |
|  | - Improved long-term health or fitness | 19 |
|  | - Meeting people | 16 |
|  | - Other (please state)   ‘Other’ response: *‘patients being in control of their treatment’* | 1 |
| What do you think the most important benefit of Prehab4Cancer is for patients? (tick one) | - Improved fitness | 7 |
|  | - Quicker recovery post-surgery | 5 |
|  | - Fewer complications post-surgery | 4 |
|  | - Improved long-term physical activity levels | 2 |
|  | - Improved long-term health or fitness | 3 |
|  | - Meeting people | 0 |
|  | - Other (please state) | 0 |
| Have you received any feedback about Prehab4Cancer from patients? | - Yes | 14 |
|  | - No | 9 |
| What do you think might help patients to take part in Prehab4Cancer? (please tick all that apply) | - A buddy system | 10 |
|  | - Travel assistance | 17 |
|  | - Support with commitments (e.g. caregiving, work) | 9 |
|  | - Correspondence with their employer | 6 |
|  | - Education about benefits of prehab | 10 |
|  | - Financial incentives | 4 |
|  | - Treatment options being restricted if they do not engage | 2 |
|  | - Other (please state)   ‘Other’ response: *‘it needs to be described as part of their treatment’* | 1 |
